# Equivalent Systemic Exposure to Fluticasone Propionate/Salmeterol Following Single Inhaled Doses from Advair Diskus and Wixela Inhub: Results of Three Pharmacokinetic Bioequivalence Studies

**DOI:** 10.1089/jamp.2019.1537

**Published:** 2020-01-30

**Authors:** Scott Haughie, Richard Allan, Nolan Wood, Jon Ward

**Affiliations:** ^1^ Mylan Pharma UK Ltd., Sandwich, United Kingdom.; ^2^ Certara UK Ltd., London, United Kingdom.

**Keywords:** Advair Diskus, asthma, COPD, fluticasone propionate, pharmacokinetic bioequivalence, salmeterol, Wixela Inhub

## Abstract

***Background:*** Wixela^®^ Inhub^®^ was developed to deliver inhaled fluticasone propionate/salmeterol (FP/S) combination as a substitutable generic equivalent to Advair^®^ Diskus^®^. These studies aimed to confirm the pharmacokinetic bioequivalence (BE) of FP/S after single doses of Wixela Inhub (test [T]) and Advair Diskus (reference [R]).

***Methods:*** Three open-label, randomized, two-way crossover, single-dose studies in healthy subjects (*N* = 66 each) compared the systemic exposure of FP and salmeterol after inhalation from three dose strengths of FP/S (100/50, 250/50, or 500/50 μg) delivered from T and R. Primary BE endpoints were the area under the plasma concentration-time curve from time = 0 to the last measurable concentration (AUC_(0-t)_) and the maximum observed plasma concentration (C_max_) for both FP and S. The BE acceptance criteria specified that the 90% confidence intervals (CIs) of the geometric mean T/R ratios for AUC_(0-t)_ and C_max_ can be contained within 0.80–1.25 for both FP and salmeterol.

***Results:*** Wixela Inhub met the acceptance criteria for BE for FP and salmeterol at each dose strength. Estimated AUC_(0-t)_ and C_max_ geometric mean ratios (T/R [90% CI]) for FP were, respectively, 1.04 (1.00–1.08) and 0.92 (0.87–0.96) for 100/50 μg FP/S, 1.07 (1.02–1.13) and 1.01 (0.95–1.07) for 250/50 μg, and 0.97 (0.92, 1.00) and 0.90 (0.86–0.93) for 500/50 μg. Estimated AUC_(0-t)_ and C_max_ ratios for salmeterol were, respectively, 1.08 (1.04–1.11) and 1.00 (0.94–1.04) for 100/50 μg FP/S, 1.03 (0.99–1.07) and 0.93 (0.87–1.00) for 250/50 μg, and 1.00 (0.96–1.04) and 0.86 (0.81–0.91) for 500/50 μg. FP/S at all doses via both T and R was comparably well tolerated.

***Conclusions:*** Wixela Inhub was bioequivalent to Advair Diskus at all three dose strengths for both FP and S, providing direct evidence of equivalent systemic safety and indirect evidence for equivalent pulmonary deposition.

## Introduction

Acombination of oral inhaled corticosteroids (ICS) and long-acting β_2_-adrenergic agonists (LABAs) is recommended for patients with asthma not controlled with ICS alone and for patients with chronic obstructive pulmonary disease (COPD) at high risk of exacerbations.^(1–4)^ Fluticasone propionate/salmeterol (FP/S) dry powder inhaler is a widely prescribed ICS/LABA fixed-dose combination drug, marketed in the United States as Advair^®^ Diskus^®^ (GlaxoSmithKline, Research Triangle Park, NC). Advair Diskus is available in three strengths, described according to the variable nominal FP dose and acknowledging the fixed 50 μg nominal dose of salmeterol base in each strength in μg: 100/50, 250/50, and 500/50. All three strengths are licensed for the twice-daily treatment of adult and adolescent asthma, the 100/50 μg strength for the management of pediatric asthma (≥4 years), and the 250/50 μg strength for the treatment of COPD.

With the expiration of U.S. patent protection for Advair Diskus in 2016,^(5)^ generic versions of the drug are progressing toward approval of an abbreviated new drug application (ANDA) by the U.S. Food and Drug Administration (FDA).^(6,7)^ The most advanced generic is Wixela^®^ Inhub^®^ (previously known as MGR001; Mylan, Inc., Canonsburg, PA), which delivers FP/S from a novel multidose dry powder inhaler (Inhub^®^ device, previously known as CRC749).^(8,9)^ A clinical development program for Wixela Inhub has been completed, and an ANDA has recently been approved.^(10)^ As part of the clinical development plan for a substitutable generic equivalent of FP/S, the FDA requires the conduct of a pharmacokinetic (PK) bioequivalence (BE) study for each of the dose strengths approved for Advair Diskus.^(11)^ Here, we report the results of three PK BE studies conducted in support of the development of Wixela Inhub.

The studies were all conducted in healthy male and female volunteers, with one study for each at the 100/50, 250/50, and 500/50 μg FP/S dose strengths. The objective of each study was to confirm the systemic PK BE of FP and salmeterol after oral inhalation of single doses of Wixela Inhub and Advair Diskus.

## Materials and Methods

In this article, “test product” (T) and “reference product” (R)^(11)^ are defined as follows: T is Wixela Inhub (FP/S administered via the Inhub device), R is Advair Diskus. Both products contained 60 premetered individual doses. Each dose of Advair Diskus comprised a white powder mix of micronized FP (100, 250, or 500 μg) and micronized salmeterol xinafoate salt (72.5 μg, equivalent to 50 μg of salmeterol base) in a 12.5 mg of formulation containing lactose monohydrate (as an excipient). The formulation contained within Wixela Inhub is qualitatively and quantitatively equivalent to that contained within Advair Diskus in terms of both active (FP and salmeterol [as xinafoate]) and inactive (lactose monohydrate) ingredients.

### Study design and conduct

Three open-label, randomized, two-way crossover studies were conducted at a single clinical center in the United States between April 2015 and July 2017, each under a separate protocol. Each study compared the systemic exposure of FP and salmeterol after FP/S administration of T and R at one of the three Advair Diskus dose strengths (FP/S 100/50, 250/50, or 500/50 μg) authorized in the United States. Study 1 evaluated FP/S 100/50 μg, study 2 evaluated FP/S 250/50 μg, and study 3 evaluated FP/S 500/50 μg. The objective of each study was to confirm the PK BE of both FP and salmeterol after oral inhalation of single doses of T and R.

The studies conformed to appropriate ethical guidelines and were conducted in accordance with the principles of the International Conference on Harmonisation of Technical Requirements for Registration of Pharmaceuticals for Human Use guideline for good clinical practice^(12)^ and the code of ethics of the World Medical Association's Declaration of Helsinki.^(13)^ Each study protocol was approved by an appropriate institutional review board, and all patients provided written informed consent.

### Study subjects and treatments

Each study was conducted in 66 healthy male and female subjects who received single orally inhaled doses of both T and R, one per study period, with a minimum 7-day washout in between. Subjects were excluded if they had used any prescription or nonprescription drugs within 7 days of the start of the study, had abnormal lung function (forced expiratory volume in 1 second <80% of predicted), or were current smokers, ex-smokers who had given up smoking for <6 months, and/or had a smoking history of ≥10 pack-years.

Each study had an identical two-treatment, two-period crossover design (2 × 2), with subjects randomized equally to each treatment sequence. To obtain adequate plasma FP and salmeterol levels, each FP/S dose strength was administered as three inhalations, resulting in total FP/S doses of 300/150 μg (study 1), 750/150 μg (study 2), and 1500/150 μg (study 3). This was implemented to ensure that both FP and salmeterol were detectable for at least three half-lives for each analyte after dosing, thus allowing an appropriate estimation of the PK parameters. In addition, the use of three inhalations was expected to reduce variability and ensure a variation coefficient (CV) of <30% for all of the BE endpoints. Based on the performance of the reference product determined in an exploratory PK study, a prospective agreement was obtained from the US FDA that the use of three inhalations was appropriate based on their stated requirement that the dose chosen should comprise the “Minimum number of inhalations that is sufficient to characterize a PK profile by using a sensitive analytical method.”^(11)^ To ensure consistent dosing, subjects received inhalation training on day −1 and day 1 (before treatment). Treatments were administered after an 8-hour fast, and standard meals were provided at ∼4 and 9–10 hours postdose and appropriate times thereafter. Water was allowed *ad libitum* throughout the study except during the period from 1 hour before dose through to 1 hour postdose.

### PK assessments and endpoints

For each study, plasma samples were obtained for each treatment period before dosing and at 2, 5, 10, 15, 20, 30, and 45 minutes and 1, 1.5, 2, 3, 4, 6, 8, 12, 24, 36, and 48 hours postdose. Sampling started at 2 minutes postdose to ensure that peak plasma concentrations of salmeterol were adequately captured, and continued up to 48 hours postdose to ensure coverage of at least three times the terminal elimination half-life (T_1/2_) estimates of the FP and salmeterol components. Drug concentrations were analyzed using validated high-performance liquid chromatography tandem mass spectrometry with a lower limit of quantification of 1 pg/mL.

Primary PK endpoints were area under the plasma concentration-time curve from time = 0 to the last measurable plasma drug concentration (AUC_(0-t)_) and maximum observed plasma drug concentration (C_max_) for both FP and salmeterol. PK parameters were derived by using noncompartmental methods using Phoenix^®^ WinNonlin^®^ (version 6.3; Certara L.P. [Pharsight], St. Louis, MO). Safety assessments included adverse events (AEs), electrocardiograms, vital signs (blood pressure and pulse rate), cardiac telemetry, and laboratory safety tests.

### Statistical methods

The safety population comprised all randomized subjects who received at least one treatment. PK parameters were calculated for all subjects who completed at least one treatment period, and those subjects who had calculable values for at least one of the primary PK parameters in at least one period were included in the PK parameter set. The statistical analysis of BE was conducted using a predefined PK analysis set (subjects who completed both treatment periods, had calculable values for at least one of the primary endpoints in both periods, and did not experience any protocol deviations or AEs that would affect PK).

Sample size calculations were based on salmeterol C_max_, since from previous Mylan studies (data on file), this PK parameter exhibits a greater within-subject standard deviation (WSD) than salmeterol AUC_(0-t)_, FP C_max_, or FP AUC_(0-t)_ (values for salmeterol C_max_ WSD in the range 0.22–0.28 on the natural log scale). Using a WSD of 0.22 and a one-sided significance level α = 0.05, sample size calculations indicated that 62 subjects in the PK analysis set gave 90% power (true difference in means of log[0.9]), and a WSD of 0.28 gave 80% power (true difference in means of log[1.1]) to demonstrate BE. Thus, a total of 66 subjects were randomized in each study to give at least 62 in the PK analysis set.

Primary endpoints (AUC_(0-t)_ and C_max_) were analyzed by using analysis of variance (ANOVA), allowing for variation due to sequence, subject within sequence, period, and treatment. The analysis was performed on the natural log scale. Least-squares mean differences (plus standard errors and 90% confidence intervals [CIs]) were produced on the log scale and exponentiated to give ratios of geometric means and associated 90% CIs on the original scale. To demonstrate BE at each dose strength,^(11)^ the 90% CIs of the T to R geometric mean ratios for AUC_(0-t)_ and C_max_ were each required to be wholly contained within the interval 0.80–1.25 (i.e., 80%–125%) for both the FP and salmeterol components. PK parameters and AEs were summarized by using descriptive statistics. All statistical analyses were conducted by using SAS^®^ version 9.3 (Cary, NC).

## Results

### Subjects

**FIG. 1.**
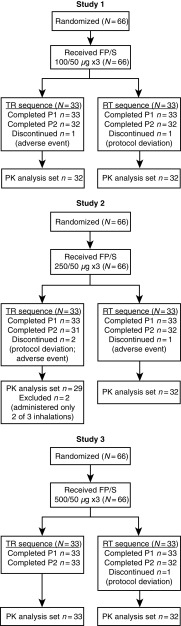
Subject disposition.

For each of the three studies (*N* = 66 for each study), all subjects completed treatment period 1 and were analyzed for safety and PK parameters; of the 198 randomized subjects across the three studies, 192 subjects also completed treatment period 2 and 190 were included in the PK analysis set (Fig. 1). Of the six subjects who did not complete treatment period 2, three subjects discontinued due to an AE and three subjects discontinued due to protocol deviations. Key baseline characteristics (age, body mass index, and tobacco history) were comparable across the three studies (Table 1).

**Table 1.** Subject Baseline Characteristics (Safety Population)

	Study 1**FP/S 100/50 μg (*N* = 66)	Study 2**FP/S 250/50 μg (*N* = 66)	Study 3**FP/S 500/50 μg (*N* = 66)
Age, *n* (%)			
Females	29 (43.9)	36 (54.5)	42 (63.6)
Males	37 (56.1)	30 (45.5)	24 (36.4)
Age, mean (range), years	33.8 (19–53)	37.7 (18–55)	35.7 (19–53)
Race, *n* (%)			
White	40 (60.6)	51 (77.3)	47 (71.2)
Black or African American	23 (34.8)	14 (21.2)	17 (25.8)
Other	3 (4.5)	1 (1.5)	2 (3.0)
BMI, mean ± SD, kg/m^2^	26.3 ± 2.7	26.3 ± (3.3)	25.8 ± 2.5
Tobacco history, *n* (%)			
Never used	54 (81.8)	57 (86.4)	58 (87.9)
Past use of tobacco	12 (18.2)	9 (13.6)	8 (12.1)

BMI, body mass index; FP/S, fluticasone propionate/salmeterol; SD, standard deviation.

### PK BE assessments

#### Fluticasone propionate

**FIG. 2.**
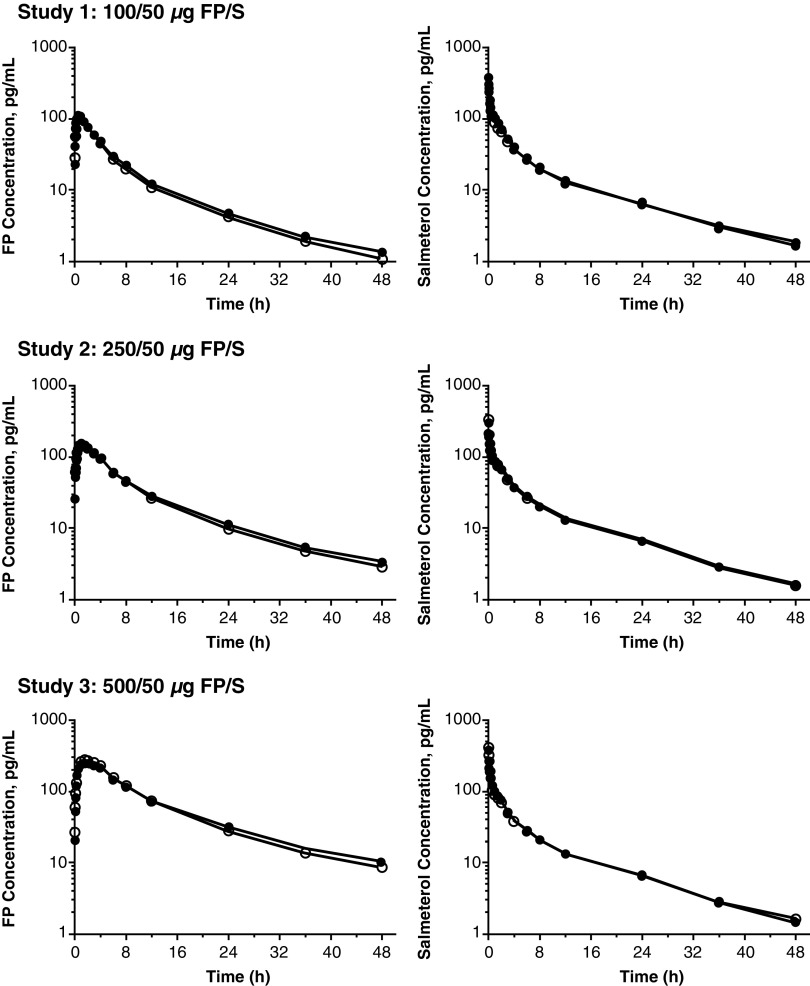
Plasma FP (left panels) and plasma salmeterol (right panels) versus time data after administration of FP/S to healthy subjects (T [closed circles] or R [open circles]) in studies 1, 2, and 3. Data presented are arithmetic mean plasma concentration (semilog scale) (*n* = 62–66). FP/S, fluticasone propionate/salmeterol.

In each study, the plasma FP concentration versus time data for T and R were comparable (Fig. 2, left panels); thus, the FP PK parameters for T and R were also comparable (Table 2). FP was rapidly absorbed, with T_max_ values ranging from 0.75 hours (study 1) to 1.5 hours (study 3). Mean C_max_ values were dose–dependent, increasing from 109 pg/mL (study 1) to 290 pg/mL (study 3). Mean total systemic FP exposure (AUC_(0-t)_) was similarly dose dependent, increasing from 609 pg•h/mL (study 1) to 2919 pg•h/mL (study 3). Mean T_1/2_ values were similar across each dose strength, ranging from 9.95 hours (study 1) to 12.23 hours (study 3).

**Table 2.** Fluticasone Propionate and Salmeterol Pharmacokinetic Parameters by Study (Pharmacokinetic Parameter Set)

	Study 1		Study 2		Study 3	
Treatment^*a*^	FP/S 100/50 μg		FP/S 250/50 μg		FP/S 500/50 μg	
Device	T (*n* = 65)	R (*n* = 65)	T (*n* = 65)	R (*n* = 64)	T (*n* = 65)	R (*n* = 66)
FP PK parameters						
T_max_, h	0.75 (0.08–1.50)	0.75 (0.08–1.50)	1.00 (0.08–3.00)	1.00 (0.33–3.01)	1.50 (0.33–4.00)	1.50 (0.33–4.00)
C_max_, pg/mL	109.7 ± 36.2	118.6 ± 35.5	170.0 ± 53.7	173.6 ± 56.4	261.6 ± 69.1	290.9 ± 74.0
AUC_(0-t)_, pg**·**h/mL	638 ± 201	609 ± 179	1298 ± 418	1237 ± 379	2851 ± 970	2919 ± 831
T_1/2_, h	10.18 ± 2.46^b^	9.95 ± 2.77^d^	11.24 ± 1.80^e^	10.38 ± 1.71^f^	12.23 ± 2.68^c^	10.57 ± 1.90
Salmeterol PK parameters						
T_max_, h	0.08 (0.03–1.50)	0.08 (0.03–2.00)	0.08 (0.04–1.01)	0.08 (0.05–2.01)	0.08 (0.03–1.00)	0.08 (0.03–1.50)
C_max_, pg/mL	385.4 ± 162.8	379.3 ± 143.9	319.5 ± 137.8	352.8 ± 158.1	376.6 ± 181.3	418.0 ± 145.8
AUC_(0-t)_, pg**·**h/mL	727 ± 223	677 ± 245	700 ± 340	686 ± 324	724 ± 297	708 ± 236
T_1/2_, h	11.87 ± 1.54^c^	12.21 ± 1.93	11.55 ± 1.71	11.66 ± 1.86^e^	11.21 ± 2.01	11.56 ± 1.86^g^

Data are shown as arithmetic mean ± standard deviation for all parameters except T_max_, which is shown as median (range).

^a^ Three inhalations in each study, resulting in total FP/S doses of 300/150 μg (study 1), 750/150 μg (study 2), and 1500/150 μg (study 3).

^b^ *n* = 62.

^c^ *n* = 64.

^d^ *n* = 60.

^e^ *n* = 63.

^f^ *n* = 59.

^g^ *n* = 65.

AUC_(0-t)_, area under the concentration-time curve from time = 0 to the last measurable concentration; C_max_, maximum plasma concentration; FP, fluticasone propionate; FP/S, fluticasone propionate/salmeterol; h, hours; PK, pharmacokinetic; R, reference product (Advair^®^ Diskus^®^); T, test product (Wixela^®^ Inhub^®^); T_1/2_, terminal elimination half-life; T_max_, time to maximum plasma concentration.

For each of the FP/S dose strengths, the geometric mean T/R ratios and 90% CIs were between 0.80 and 1.25 for FP AUC_(0-t)_ and FP C_max_ (Table 3), indicating that T and R were bioequivalent for the FP component.

**Table 3.** Bioequivalence of Fluticasone Propionate and Salmeterol (Pharmacokinetic Analysis Set)

Treatment^*a*^	AUC_(0-t)_ (pg·h/mL)	AUC_(0-t)_ T/R ratio (90% CI)^*a*^	C_max_ (pg/mL)	C_max_ T/R ratio (90% CI)^*b*^
Study 1: FP/S 100/50 μg (*n* = 64)				
Fluticasone propionate				
T	600.3	1.04 (1.00–1.08)	103.7	0.92 (0.87–0.96)
R	576.4		112.9	
Salmeterol				
T	696.4	1.08 (1.04–1.11)	347.7	1.00 (0.94–1.04)
R	644.9		348.3	
Study 2: FP/S 250/50 μg (*n* = 61)				
Fluticasone propionate				
T	1251	1.07 (1.02–1.13)	164.2	1.01 (0.95–1.07)
R	1164		162.7	
Salmeterol				
T	641.2	1.03 (0.99–1.07)	296.2	0.93 (0.87–1.00)
R	623.3		317.4	
Study 3: FP/S 500/50 μg (*n* = 65)				
Fluticasone propionate				
T	2689	0.97 (0.92–1.00)	252.8	0.90 (0.86–0.93)
R	2783		281.8	
Salmeterol				
T	672.3	1.00 (0.96–1.04)	334.9	0.86 (0.81–0.91)
R	670.8		388.6	

Data presented as natural-log transformed geometric mean (based on least squares mean).

^a^ Three inhalations were administered in each study, resulting in total FP/S doses of 300/150 μg (study 1), 750/150 μg (study 2), and 1500/150 μg (study 3).

^b^ T and R were bioequivalent if the 90% CIs of the T to R geometric mean ratio were >0.80 and <1.25.

AUC_(0-t)_, area under the concentration-time curve from time = 0 to the last measurable concentration; CI, confidence interval; C_max_, maximum plasma concentration; FP/S, fluticasone propionate/salmeterol; PK, pharmacokinetic; R, reference product (Advair^®^ Diskus^®^); T, test product (Wixela^®^ Inhub^®^).

#### Salmeterol

In each study, the plasma salmeterol concentration versus time data for T and R were comparable (Fig. 2, right panels); thus, the salmeterol PK parameters for T and R were also comparable (Table 2). Salmeterol was very rapidly absorbed, with a T_max_ value of 5 minutes across the three studies. Mean C_max_ values were consistent across studies, ranging from 319 pg/mL (study 2) to 418 pg/mL (study 3). Mean AUC_(0-t)_ was similarly consistent, ranging from 677 pg•h/mL (study 1) to 724 pg•h/mL (study 3). Mean T_1/2_ values were also consistent, ranging from 11.21 hours (study 3) to 12.21 hours (study 1).

For each of the FP/S dose strengths, the 90% CI of the geometric mean T/R ratios for salmeterol AUC_(0-t)_ and salmeterol C_max_ were between 0.80 and 1.25 (Table 3), indicating that T and R were bioequivalent for the salmeterol component.

### Safety results

FP/S was well tolerated for both T and R in all studies with no clinically significant changes in electrocardiograms, vital signs (blood pressure and pulse rate), cardiac telemetry, or laboratory safety tests. AEs were generally mild and occurred with similar frequencies in T- and R-treated subjects in all studies (Table 4). The most commonly reported AE was headache. One subject experienced a serious AE, classified as dyspnea of moderate severity, that occurred after completion of treatment with R in study 1 (100/50 μg dose strength) and was considered by the investigator not to be treatment related. One subject treated with T in study 1 experienced an upper respiratory tract infection of mild severity (considered by the investigator to be unrelated to treatment) that led to discontinuation.

**Table 4.** Safety Overview (Safety Population)

	Study 1		Study 2		Study 3	
Treatment^*a*^	100/50 μg		250/50 μg		500/50 μg	
Device	T (*n* = 65)	R (*n* = 65)	T (*n* = 65)	R (*n* = 64)	T (*n* = 65)	R (*n* = 66)
Subjects reporting ≥1 treatment-emergent AE, *n* (%)	4 (6.2)	9 (13.8)	11 (16.9)	5 (7.8)	4 (6.2)	0
Subjects reporting ≥1 serious AE, *n* (%)	0	1 (1.5)^b^	0	0	0	0
Subjects reporting ≥1 AE leading to study discontinuation, n (%)	1 (1.5)^c^	0	1 (1.5)	1 (1.6)	0	0
Most commonly reported AEs^d^, *n* (%)						
Headache	1 (1.5)	2 (3.1)	0	2 (3.1)	0	0
Dizziness	0	1 (1.5)	1 (1.5)	0 (0.0)	0	0
Vessel puncture site pain	0	0	3 (4.6)	2 (3.1)	0	0
Presyncope	1 (1.5)	1 (1.5)	2 (3.1)	0 (0.0)	0	0

^a^ Three inhalations were administered in each study, resulting in total FP/S doses of 300/150 μg (study 1), 750/150 μg (study 2), and 1500/150 μg (study 3).

^b^ One case of moderate dyspnea was reported 2 days 7 hours after FP/S administration and after completion of all study procedures; this AE was considered by the investigator as not related to study treatment.

^c^ One nonserious case of mild upper respiratory tract infection led to discontinuation of this subject before period 2; this AE was considered by the investigator as not related to study treatment.

^d^ AEs (preferred terms) reported by two or more subjects with any treatment.

AE, adverse event; FP/S, fluticasone propionate/salmeterol; R, reference product (Advair^®^ Diskus^®^); T, test product (Wixela^®^ Inhub^®^).

## Discussion

Wixela Inhub is being developed as a generic equivalent to Advair Diskus. These studies, one for each of the three authorized Advair Diskus dose strengths, confirmed the PK BE of both FP and salmeterol components after oral inhalation of single doses of Wixela Inhub and Advair Diskus. For the FP/S 100/50 μg, 250/50 μg, and 500/50 μg dose strengths, BE criteria were fully met for both FP and salmeterol for each primary endpoint (AUC_(0-t)_ and C_max_), in accordance with regulatory guidance.^(11)^

PK parameters for FP and salmeterol after treatment with FP/S were consistent with published data on Advair Diskus^(14–18)^; however, the higher total FP/S doses of the current studies (300/150, 750/150, and 1500/150 μg) complicate a direct comparison of PK parameters with those from previous studies, which used lower total doses (100/50 and 250/50 μg). The use of higher total FP/S doses (three inhalations) in the current studies allowed for a thorough understanding of the PK profile of both FP and salmeterol from both Wixela Inhub and Advair Diskus, including an assurance that the plasma concentrations of each analyte were readily detectable to at least 12 hours postdose, and thus enabled a complete comparison of both T and R. In addition, the use of three inhalations of FP/S is associated with less variability of exposure, particularly for the 100/50 μg strength (within subject CV <30%), compared with the same dose administered with one inhalation (within subject CV >30% [Mylan data on file]).

Within each dose strength, FP and salmeterol PK parameters for T and R were similar. Peak plasma concentrations of FP and salmeterol occurred at 1–2 hours and 5 minutes, respectively, as previously reported.^(18,19)^ The mean T_1/2_ estimated for FP in these studies (11.01 hours) was longer than some previous reports (4.7 hours^(17)^ and 7.8 hours^(18)^) and similar to other estimates (11.4 hours^(20)^ and 12.5 hours^(21)^). This increased T_1/2_ could be a reflection of the fact that in this study three inhalations of FP/S were given, which meant that concentrations were sufficient to allow thorough characterization of the terminal phase. Therefore, the reported half-lives in this study are considered accurate.

Burmeister-Getz et al.^(21)^ have reported that for Advair Diskus 100/50 μg, the inherent variability of R means it is not likely that a 2 × 2 comparison of a generic FP/S with Advair Diskus would achieve BE according to current FDA standards; our studies are counter to this position. It is recognized that variability exists in the PK response to Advair Diskus; however, if appropriate *in vitro* assessments such as fine particle mass (FPM) are performed across a large range of batches of Advair Diskus, it is possible to characterize the population of Advair Diskus batches. While remaining within the pharmaceutical specification for Advair Diskus, batches of R that are near the extremes of the distribution exist (e.g., a batch that has high FPM and another batch that has low FPM), and if such batches are compared, they can be shown not to be bioequivalent in a standard human PK study (Mylan data on file). However, if the comparison between the same two batches is corrected for the FPM content, they can be shown to be bioequivalent by using the same study data.

The Burmeister-Getz et al.^(21)^ study did not report the key *in vitro* characteristics of the batches of Advair Diskus used, reporting only the age of the batches, which is not an adequate indicator of pharmaceutical performance of the product. However, if batches of both T and R are well matched for *in vitro* parameters, and the R batch is representative of the Advair Diskus population, then PK BE can be achieved as demonstrated in these studies for all dose strengths of FP/S.

In addition, Burmeister-Getz et al.^(19)^ have reported that variability of product batches may lead to an increase in type 1 error rate beyond the accepted 5% level by using the standard 2 × 2 crossover design. The assumption underlying this finding is that batches of T and R included in a PK study are chosen entirely at random (i.e., selected from any point of the distribution for each product). The source of the inflated type 1 error is the overlap of the distribution of the individual T and R batches around the respective T and R averages. In general, the greater the interbatch variability, the wider this overlap, and the greater the chance of the erroneous finding that batches are bioequivalent, even if the product averages are not bioequivalent.

Recently, the same authors^(22)^ presented the results of a PK study utilizing a multibatch design, which demonstrated BE for OT329 Solis 100/50 μg versus Advair Diskus 100/50 μg. This study design was not consistent with FDA guidance^(11)^ but represented a novel, unprecedented approach to demonstrating PK BE, which was presumably developed to resolve the authors' concerns about the properties of standard PK BE designs raised in earlier publications. Although this and other multibatch approaches are still very much in their infancy,^(21)^ we believe that the standard PK BE designs we have utilized, in combination with a thorough understanding of the *in vitro* characteristics that drive interbatch variability of both T and R, and well-established statistical analysis methods, still represent a robust and reliable assessment of the PK BE of FP/S combination products, in line with FDA guidance.

The recruitment of healthy subjects instead of subjects with asthma or COPD enabled a comprehensive assessment of systemic exposure of FP and salmeterol without the potential confounding factors such as variable and compromised pulmonary function or the use of concomitant medications, all of which could have a direct influence on the absorption, distribution, metabolism, and excretion of the study drugs. As the use of healthy subjects allows for consistent disease status and no requirement to modulate a patient's treatment regime, it is possible to conduct crossover design studies that enable a within-subject comparison of exposure, and thus the variability of a study is reduced substantially. Likely for these reasons, the use of healthy volunteers is reflected in regulatory guidance,^(11)^ and healthy volunteers were recently used for similar BE studies.^(14,23)^ In addition, a meta-analysis^(24)^ demonstrated that although the apparent bioavailability of FP in healthy subjects is greater by ≈2-3-fold versus asthma subjects, there is conservation of the relative bioavailability when comparing the delivery of FP from different inhalation devices (i.e., the difference in exposure for FP delivered from Diskhaler^®^ [GlaxoSmithKline, Research Triangle Park, NC]) and Diskus^®^ was ∼15% in both asthmatic and healthy subjects). This conservation of relative bioavailability suggests that if generic FP/S demonstrates PK BE to Advair Diskus in healthy subjects, it would likely also demonstrate PK BE in a patient population.

As many AEs associated with FP and salmeterol are related to systemic exposure to these products, demonstrating equivalent exposure indirectly demonstrates that a generic ICS/LABA should have a safety profile generally similar to the originator's product. This is particularly true of orally inhaled products that have poor systemic bioavailability such as FP, as the measured systemic exposure would be almost entirely related to the lung dose of the drug. The AEs observed for both T and R in the current studies were consistent in nature and frequency with those reported for Advair Diskus.^(18)^ All AEs were mild or moderate in severity and of low incidence compared with the most commonly reported AEs according to the Advair Diskus prescribing information.^(18)^

In conclusion, our investigation confirmed that Wixela Inhub demonstrated systemic PK BE to Advair Diskus at all FP/S dose strengths using a consistent approach, with standard study designs for all FP/S dose strengths. Moreover, a study using clinical efficacy endpoints recommended by the FDA^(11)^ has recently been completed as part of the Wixela Inhub clinical development plan (NCT02245672). That study demonstrated local (lung) BE of Wixela Inhub and Advair Diskus in patients with asthma based on the effects of both active treatments on lung function endpoints (forced expiratory volume in 1 second) measured after the first dose and 4 weeks of dosing that were both superior to placebo and statistically equivalent to each other.^(25)^ Wixela Inhub, therefore, represents a substitutable generic equivalent FP/S treatment option for subjects with asthma whose symptoms are uncontrolled with ICS alone and for subjects with COPD at high risk of exacerbations.

## Notes

Reviewed by:

Ronald Wolff
